# Synphilin-1 Is Essential for Cytoskeletal Integrity of Brain Ventricular Cilia and Mitochondrial Proteostasis

**DOI:** 10.3390/ijms27083499

**Published:** 2026-04-14

**Authors:** Malik Farhoud, Ankit Kumar Shah, Nicole Pavoncello, Haya Hamza, Fatimah Abd Elghani, Vered Shani, Michal Toren-Hershkoviz, Sofia Zaer, Galit Saar, Lihi Shaulov, Zagorka Vitic, Claude Brodski, Inon Maoz, Salman Zubedat, Avi Avital, Hazem Safory, Simone Engelender

**Affiliations:** 1 Department of Biochemistry, The Ruth and Bruce Rappaport Faculty of Medicine, Technion-Israel Institute of Technology, Haifa 31096, Israel; malik.far.96@gmail.com (M.F.); nicole.p@campus.technion.ac.il (N.P.); hsafory@technion.ac.il (H.S.); 2 Department of Physiology and Cell Biology, School of Brain Sciences and Cognition, Faculty of Medicine, Ben-Gurion University of Negev, Be’er-Sheva 84105, Israel; 3 Department of Occupational Therapy, Faculty of Social Welfare and Health Sciences, University of Haifa, Haifa 3498838, Israel

**Keywords:** Parkinson’s disease, neurodegeneration, synphilin-1, microtubules, cilia, hydrocephalus

## Abstract

Parkinson’s disease (PD) is a common neurodegenerative disorder marked by progressive loss of dopaminergic neurons in the substantia nigra pars compacta and the accumulation of Lewy bodies, intracellular inclusions enriched in α-synuclein. Synphilin-1 interacts with α-synuclein, localizes to Lewy bodies, and has been implicated in inclusion formation and neuroprotection in cellular and animal models; however, its physiological function in vivo remains poorly defined. Here, we generated and characterized a synphilin-1 knockout (Sph-1 KO) mouse by targeted genetic deletion of the Sph-1 locus and performed a comprehensive phenotyping battery including behavioral testing as well as biochemical, histological, structural, and ultrastructural analyses. Sph-1 KO mice survived to nearly two years of age and showed normal body weight, lifespan, motor performance, learning and memory, anxiety-like behavior, attention, and gross brain morphology. Western blot analyses indicated that levels of α-synuclein and synaptic proteins were largely unchanged. While outer mitochondrial membrane proteins were unaffected, the mitochondrial matrix protein HSP60 was reduced, consistent with altered mitochondrial proteostasis in the absence of synphilin-1. Strikingly, histochemical analyses, magnetic resonance imaging, and electron microscopy revealed early-onset hydrocephalus in Sph-1 KO mice associated with severe loss and disorganization of motile ependymal cilia in the ventricular lining, a cell type that normally expresses high levels of synphilin-1. Ultrastructural and immunohistochemical analyses revealed disrupted ependymal architecture, mislocalization of acetylated α-tubulin to the cytoplasm, cellular swelling, and enlarged, aberrant mitochondria, whereas cortical neurons appeared largely structurally unaffected. Together, these findings identify synphilin-1 as a key regulator of microtubule organization and cytoskeletal/organelle homeostasis in ependymal cells, required to maintain motile ciliogenesis, cerebrospinal fluid flow, and ventricular integrity. This unexpected role for synphilin-1 in ciliated brain epithelia, along with a reduction in the critical mitochondrial chaperone HSP60, broadens our understanding of synphilin-1 biology and provides a new framework for its potential relevance to PD-associated pathology.

## 1. Introduction

Parkinson’s disease (PD) is one of the most prevalent neurodegenerative disorders, affecting approximately 1% of individuals over the age of 65, with incidence increasing further with age [1]. Clinically, PD is characterized by progressive motor symptoms (bradykinesia, rigidity, resting tremor, and postural instability) alongside prominent non-motor features, including autonomic dysfunction, sleep disturbances, mood changes, and cognitive decline [1]. Pathologically, PD is defined by the degeneration of dopaminergic neurons in the substantia nigra pars compacta and by the presence of Lewy bodies and Lewy neurites [2]. These intracellular inclusions are composed primarily of α-synuclein, together with vesicles, mitochondrial components, and various cytoskeletal components [3,4].

We previously identified synphilin-1 as an α-synuclein-interacting protein [5], and a rare R621C mutation in its gene has been associated with PD in specific populations [6,7]. Synphilin-1 is a large, interaction-rich protein containing ankyrin-like repeats and a central coiled-coil region, consistent with a scaffolding role in multiprotein assemblies [5]. Synphilin-1 is present within Lewy bodies [8] and promotes inclusion formation in cellular and animal models [5,9,10,11,12,13,14]. Although its physiological function remains incompletely understood, synphilin-1 has been described as neuroprotective in cellular models, where it modulates energy homeostasis and basal mitophagy [14,15,16]. Consistently, several synphilin-1 overexpression mouse models have shown variable degrees of aggregation and toxicity [10,11,17,18], and synphilin-1 can counteract deleterious effects when co-expressed with α-synuclein and pathogenic LRRK2 mutants [10,11,19,20]. Together, these findings underscore the need for further investigation into synphilin-1’s endogenous role in the brain and its potential contribution to PD-relevant pathways.

To date, the in vivo function of endogenous synphilin-1 has not been defined. To address this gap, we generated synphilin-1 knockout (Sph-1 KO) mice and applied a comprehensive phenotyping approach integrating behavioral assays with biochemical, histological, structural, and ultrastructural analyses. Elucidating synphilin-1’s physiological role may help clarify mechanisms that contribute to neurodegeneration in PD.

## 2. Results

### 2.1. Generation of Synphilin-1 Knockout Mice

**Figure 1:**
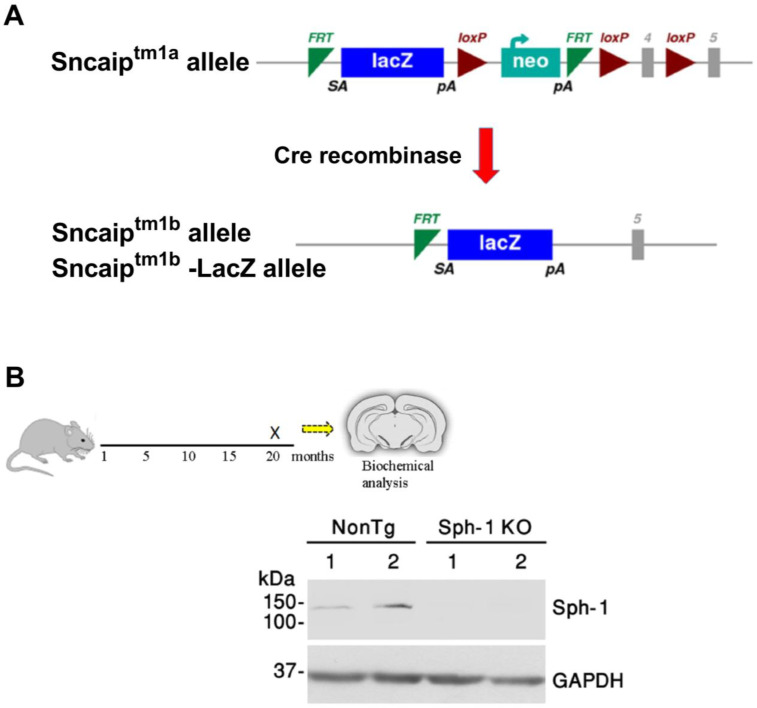
**Generation of synphilin-1 knockout mice.** (**A**) Schematic representation of the knockout strategy. The Sncaip^tm1a^ allele (upper schematic) contains a *lacZ* reporter cassette inserted into an intronic region downstream of exon 3, flanking exon 4 of the SNCAIP gene. The null Sncaip^tm1b^ allele (lower schematic) was generated from the Sncaip^tm1a^ allele by Cre-mediated recombination, resulting in deletion of exon 4 and removal of the neomycin (neo) selection cassette from the genomic locus. Exons are shown as gray boxes. FRT, FLP recombination target sites; SA, splice acceptor; lacZ, β-galactosidase reporter gene; pA, polyadenylation signal; loxP, Cre-loxP recombination sites; neo, neomycin resistance cassette; Cre, cyclization recombination enzyme. (**B**) A schematic timeline indicates that mice were sacrificed at 20 months of age for biochemical analyses. Synphilin-1 (Sph-1) protein levels in the brains of synphilin-1 knockout (Sph-1 KO) and non-transgenic (NonTg) control mice at 20 months of age were determined by immunoblot analysis using anti-synphilin-1 antibody. GAPDH was used as a loading control (**lower panel**). The image is representative of three independent Western blot analyses. For both the NonTg and Sph-1 KO groups, two animals were analyzed (*n* = 2 per group).

The physiological roles of endogenous synphilin-1, particularly in neuronal survival, remain unclear. To elucidate its in vivo functions, we generated a novel synphilin-1 knockout (Sph-1 KO) mouse line. Synphilin-1 (SNCAIP) mutant mice were obtained from the European Mouse Mutant Cell Repository (EUCOMM). The targeted Sncaip^tm1a^ allele is a conditional gene-targeting allele containing a LacZ reporter and a promoter-driven selection cassette, enabling disruption of SNCAIP while also allowing for the monitoring of its endogenous expression. To generate a constitutive knockout line, EUCOMM bred Sncaip^tm1a^ mice to a Cre-deleter strain, resulting in Cre-mediated recombination at the loxP sites flanking exon 4 and the neomycin/selection cassette. This recombination excised exon 4 together with the selection cassette, producing the Sncaip^tm1b(EUCOMM)Wtsi^ allele (EM08403) (Figure 1A). Mice were provided to us as heterozygous Sncaip^tm1b(EUCOMM)Wtsi^ and were subsequently intercrossed to generate homozygous Sncaip ^tm1b(EUCOMM)Wtsi^ mice. Animals homozygous for the exon 4-deleted allele are referred to here as Sph-1 KO mice. Western blot analysis of cortical lysates from 20-month-old mice confirmed the absence of synphilin-1 protein expression in Sph-1 KO brains (Figure 1B).

### 2.2. Synphilin-1 Knockout Alters the Levels of the Mitochondrial Matrix Chaperone HSP60

**Figure 2:**
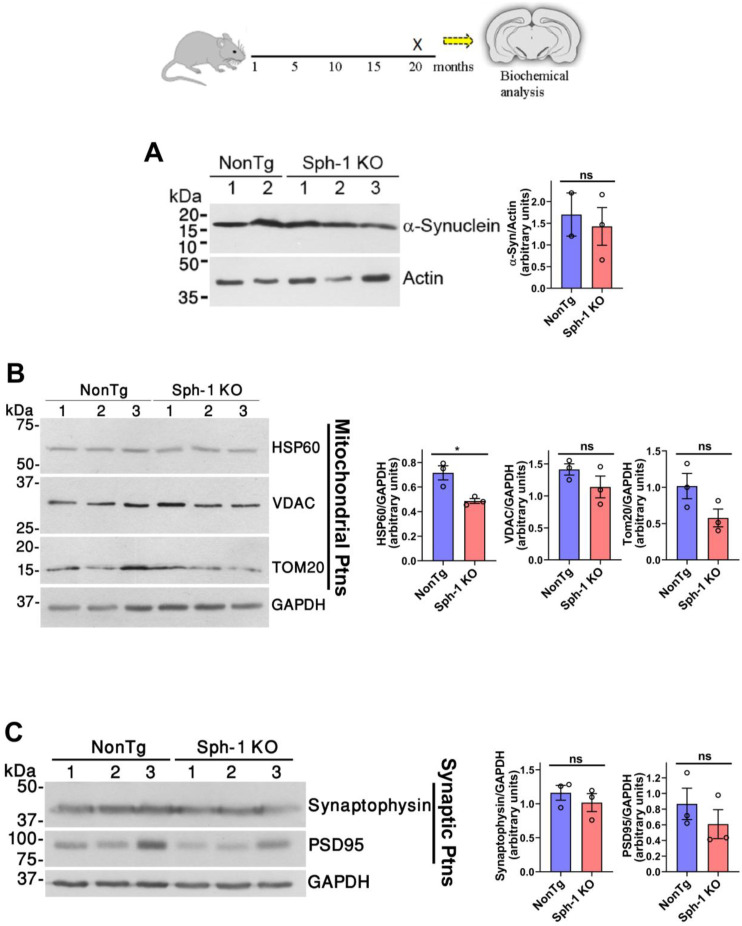
**Synphilin-1 knockout reduces the levels of the mitochondrial chaperone HSP60.** A schematic timeline indicates that mice were sacrificed at 20 months of age for biochemical analyses. Frontal cortex lysates from 20-month-old Sph1 KO and NonTg mice were analyzed by Western blotting. (**A**) The levels of α-synuclein were determined with anti-α-synuclein antibody. The lower panel depicts the loading control actin. (**B**) Levels of the mitochondrial proteins HSP60, VDAC, and TOM20 were detected using specific antibodies. GAPDH was used as the loading control. (**C**) Levels of the presynaptic and postsynaptic proteins synaptophysin and PSD95, respectively, were determined using specific antibodies. GAPDH was used as a loading control. The figures shown are representative of three independent Western blot analyses. Graphs depict the relative levels of indicated proteins in Sph-1 KO mice compared with NonTg controls. For each group, 2 or 3 animals were analyzed (*n* = 2–3 per group). Data are presented as mean ± SEM. Statistical significance was assessed using Student’s unpaired two-tailed *t*-test; * *p* ≤ 0.05 versus NonTg controls; ns, not significant.

Because synphilin-1 interacts with α-synuclein and promotes inclusion body formation [5], we examined whether loss of synphilin-1 impacts α-synuclein expression. Western blot analysis of frontal cortex lysates from 20-month-old mice revealed no significant difference in α-synuclein protein levels between non-transgenic (NonTg) and Sph-1 KO mice (Figure 2A).

Given that mitochondrial dysfunction contributes to PD pathogenesis [21,22] and that synphilin-1 has been implicated in promoting basal mitophagy [15], we assessed whether its loss affects mitochondrial protein expression. Analysis of frontal cortex lysates revealed no significant differences in the levels of the outer mitochondrial proteins’ voltage-dependent anion channel (VDAC) and TOM20 between Sph-1 KO mice and NonTg controls (Figure 2B). In contrast, the mitochondrial matrix chaperone HSP60 was reduced in Sph-1 KO mice compared with NonTg controls (Figure 2B). These findings indicate that synphilin-1 knockout does not affect major mitochondrial outer membrane components, but it does reduce the mitochondrial matrix chaperone HSP60, a key factor in maintaining mitochondrial proteostasis [23].

Because both synphilin-1 and α-synuclein localize to the presynapse [24,25], we also examined whether synphilin-1 deletion affects synaptic integrity. Analysis of cortical lysates showed no differences in the levels of the presynaptic marker synaptophysin or the postsynaptic density protein PSD95 (Figure 2C). These results indicate that synphilin-1 knockout does not alter the major molecular components of synaptic terminals.

### 2.3. Synphilin-1 Knockout Does Not Lead to Motor, Sensory, or Memory Phenotypes

**Figure 3:**
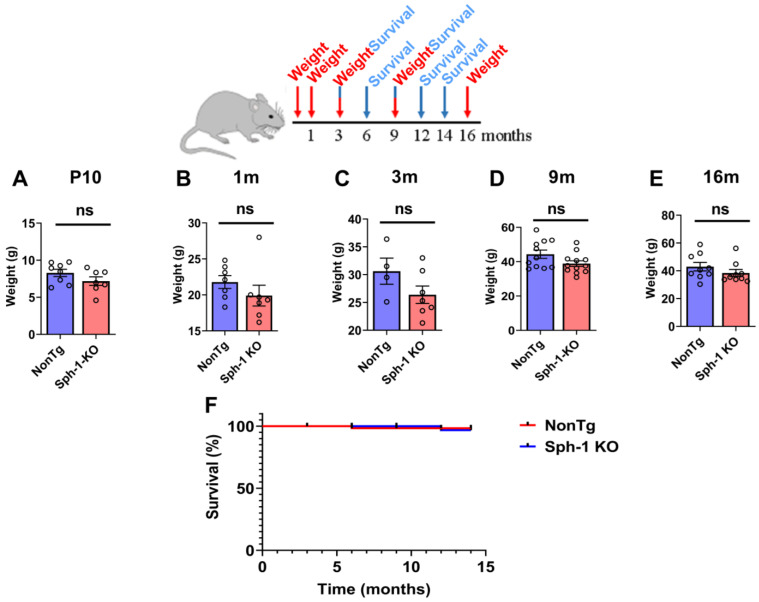
**Synphilin-1 knockout does not affect body weight or survival in mice.** A schematic timeline indicates the time points at which body weight (in red) and survival (in blue) were assessed. Accordingly, body weights of Sph-1 KO and NonTg mice were measured at 10 days (**A**), 1 month (**B**), 3 months (**C**), 9 months (**D**), and 16 months (**E**) of age. Graphs show body weight in Sph-1 KO mice relative to NonTg controls. Data are presented as mean ± SEM. For the NonTg group, body weight was measured in 4–11 mice at P10 and at 1, 3, 9, and 16 months of age. For the Sph-1 KO group, 7–12 mice were assessed at the corresponding time points. Group comparisons were performed using Student’s unpaired two-tailed *t*-test, and no significant differences were observed (*p* > 0.05). (**F**) Kaplan–Meier survival curves comparing Sph-1 KO and NonTg mice up to 14 months of age. For the NonTg group, survival was evaluated in 75–15 mice at 1, 3, 6, 9, 12, and 14 months of age. For the Sph-1 KO group, survival was evaluated in 76–16 mice at the corresponding time points. Survival distributions were compared using the log-rank test, and no significant (ns) differences were observed (*p* > 0.05).

We monitored the body weight of Sph-1 KO mice from early postnatal development (10 days old) through to late adulthood (16 months) and observed no significant differences compared with NonTg control mice (Figure 3A–E). In addition, survival analysis up to 14 months of age revealed no differences between Sph-1 KO and NonTg mice (Figure 3F).

**Figure 4:**
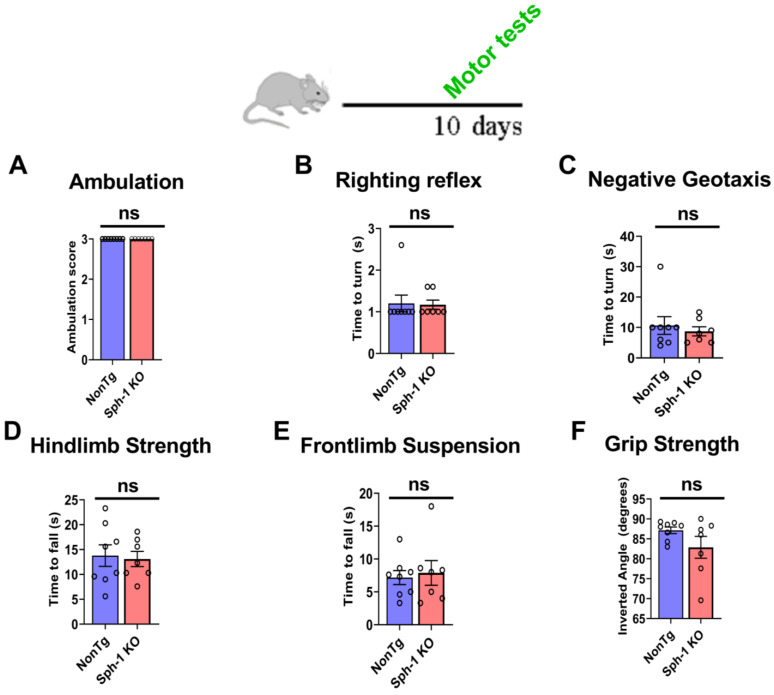
**Synphilin-1 knockout does not cause motor dysfunction in neonatal mice.** A schematic timeline indicates that motor function was evaluated in 10-day-old mice by assessing ambulation score (**A**), righting reflex (**B**), negative geotaxis (**C**), hindlimb strength (**D**), front-limb suspension (**E**), and four-paw grip strength (**F**). Graphs show motor performance of 10-day-old Sph-1 KO mice relative to age-matched NonTg controls. Motor tests were performed in 8 NonTg and 7 Sph-1 KO mice. Data are presented as mean ± SEM. Statistical comparisons were conducted using Student’s unpaired two-tailed *t*-test for each assay, and no significant (ns) differences were observed between groups (*p* > 0.05).

To characterize the behavioral phenotype of Sph-1 KO mice, we performed a comprehensive battery of behavioral assays from early postnatal stages through late adulthood. In young pups, we assessed multiple aspects of motor function, including ambulation, surface righting, negative geotaxis, hindlimb suspension, front-limb suspension, and grip strength (Supplementary Figure S1) [26]. We found that Sph-1 KO pups displayed normal crawling behavior, comparable to the NonTg controls (Figure 4A). The righting reflex was also intact, as righting times did not differ between groups (Figure 4B). Motor coordination, evaluated using the negative geotaxis test, was similarly unaffected, where Sph-1 KO pups required the same amount of time as NonTg pups to reorient upward on a 45° incline (Figure 4C). Muscle strength was evaluated through hindlimb and front-limb suspension tests. Hindlimb strength appeared normal, as the latency to fall was comparable between Sph-1 KO and NonTg pups (Figure 4D). Likewise, front-limb strength, assessed by the front-limb suspension test, showed no significant differences between groups (Figure 4E). Finally, overall limb strength was measured using a grip strength assay, in which the angle at which pups released the grip did not differ between genotypes (Figure 4F). Collectively, these findings demonstrate that Sph-1 KO pups exhibit no detectable motor deficits, indicating the absence of an early motor behavioral phenotype.

Because PD is primarily a disorder of aging, we next examined whether adult Sph-1 KO mice might exhibit a late-onset behavioral phenotype. To this end, we conducted a comprehensive battery of behavioral assays designed to assess multiple functional domains, including motor performance (pole and rotarod tests), motor coordination (rotarod test), motor asymmetry (cylinder test), motor learning (rotarod test), locomotor activity and anxiety-like behavior (open field test), hippocampal-dependent learning and memory (Morris water maze), and habituation and sensorimotor gating (acoustic startle response) (Supplementary Figure S2) [27,28].

**Figure 5:**
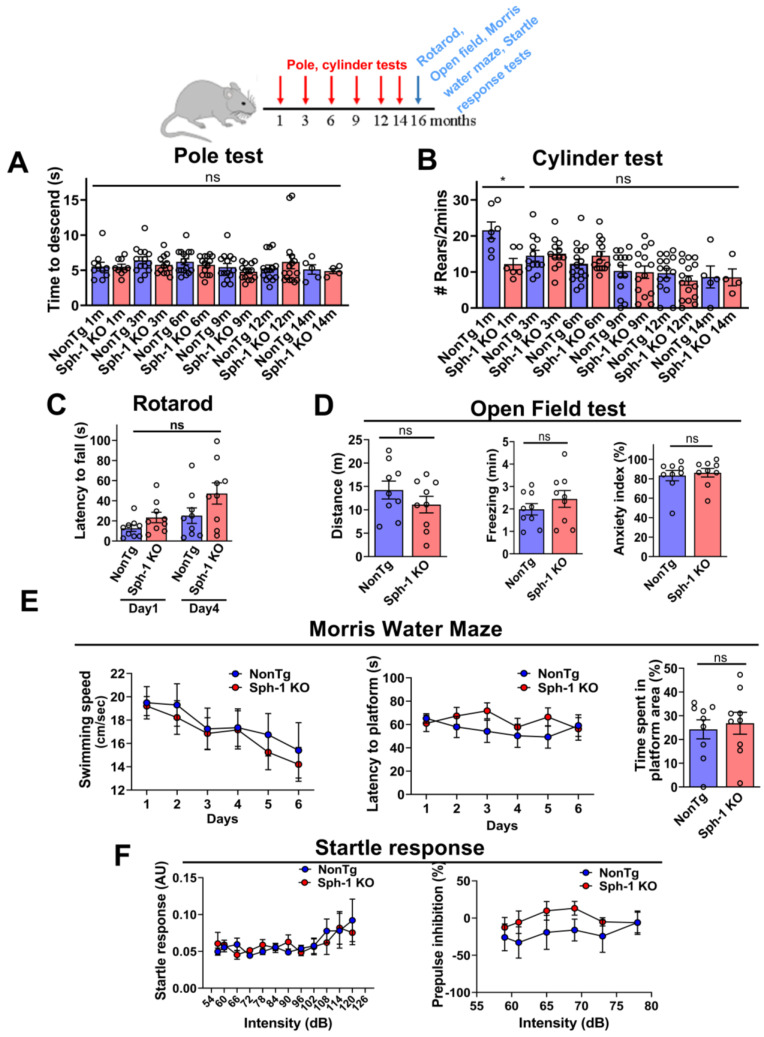
**Synphilin-1 knockout does not cause anxiety-like behavior or impair motor, learning, or sensory function in adult mice.** A schematic timeline indicates the time points at which behavioral testing were performed. Specifically, Sph-1 KO and NonTg mice (1–12 months of age) were assessed for motor performance using the pole (**A**) and cylinder (**B**) tests. Graphs show motor performance of Sph-1 KO mice relative to age-matched NonTg controls. Motor tests were performed in 5–16 NonTg and 4–16 Sph-1 KO mice. Data are presented as mean ± SEM. Statistical comparisons were conducted using Student’s unpaired two-tailed *t*-test for each time point. Except at 1 month, where a significant difference was observed (* *p* ≤ 0.05), no significant differences were detected between Sph-1 KO and NonTg controls at the other time points (*p* > 0.05). (**C**) Motor coordination and endurance at 16 months were assessed by rotarod (mean ± SEM; two-way ANOVA with Tukey’s post hoc test; *p* > 0.05). (**D**) Open field testing at 16 months assessed total distance traveled (**left**), immobility/freezing time (**middle**), and anxiety-like behavior (% freezing; **right**) (mean ± SEM; two-tailed Student’s *t*-test; *p* > 0.05). (**E**) Morris water maze at 16 months assessed swim speed (**left**), learning (latency to hidden platform; **middle**), and memory (time in target quadrant after platform removal; **right**) (mean ± SEM; two-way ANOVA with Tukey’s post hoc test for (**left**/**middle**), and Student’s unpaired two-tailed *t*-test for **right**; *p* > 0.05). (**F**) Acoustic startle at 16 months measured startle amplitude (**left**) and pre-pulse inhibition (**right**) (mean ± SEM; two-way ANOVA with Tukey’s post hoc test, *p* > 0.05). For (**C**–**F**), graphs compare Sph-1 KO mice with age-matched NonTg controls. Testing was performed in 9 NonTg and 9 Sph-1 KO mice. ns, not significant.

We first focused on motor assays that are directly relevant to basal ganglia-associated movement impairments. These included the pole test, which is sensitive to subtle motor deficits, and the cylinder test, which evaluates motor symmetry [27]. Across all ages tested (1–12 months), Sph-1 KO mice performed similarly to NonTg controls in both assays (Figure 5A,B). These results indicate that adult Sph-1 KO mice do not exhibit motor impairments, suggesting that loss of synphilin-1 does not affect overall motility or forelimb symmetry. We also evaluated motor function, motor learning, and coordination using the rotarod test. Although Sph-1 KO mice remained on the accelerating rod for slightly longer periods than NonTg controls, this difference did not reach statistical significance (Figure 5C).

We next assessed locomotor activity and anxiety-like behavior using the open field test [28]. No differences were observed between Sph-1 KO and NonTg mice in any of the measured parameters (Figure 5D). Specifically, the groups showed comparable total distance traveled (Figure 5D, left), stationary/freezing time (Figure 5D, middle), and percentage of time spent in prolonged freezing bouts, an indicator of anxiety-like behavior (Figure 5D, right). These results indicate that synphilin-1 knockout does not alter locomotor activity or anxiety levels in adult mice.

Moreover, the spatial learning and memory of the mice were evaluated using the Morris water maze [28]. Swimming speed (cm/s) did not differ between Sph-1 KO and NonTg mice (Figure 5E, left graph), consistent with the absence of motor deficits observed in the pole, cylinder, and rotarod tests (Figure 5A–C). In the acquisition phase, Sph-1 KO mice required a similar amount of time to locate the hidden platform as NonTg controls (Figure 5E, middle graph), indicating intact spatial learning. During the probe trial, both groups spent comparable time in the hidden platform area after its removal (Figure 5E, right graph), demonstrating that spatial memory is preserved in Sph-1 KO mice.

Finally, to evaluate habituation and sensorimotor gating, we assessed the startle reflex using the acoustic startle response test. Sph-1 KO mice showed normal startle responses, comparable to those of NonTg controls (Figure 5F, left graph). Moreover, pre-pulse inhibition was unchanged in Sph-1 KO mice (Figure 5F, right graph), indicating that sensorimotor gating is intact.

### 2.4. Synphilin-1 Knockout Leads to Hydrocephalus

**Figure 6:**
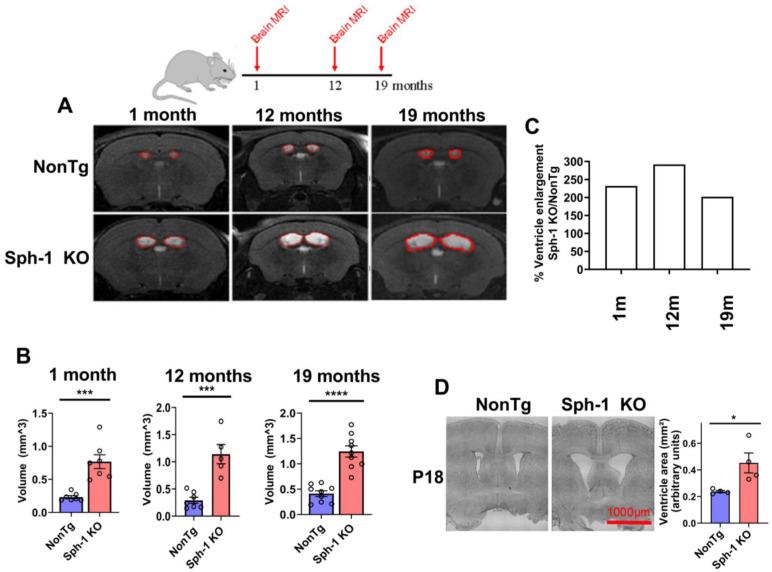
**Synphilin-1 knockout mice develop hydrocephalus.** A schematic timeline indicates the time points at which MRI scans were performed. (**A**) Representative brain MRI images from Sph-1 KO (**lower panels**) and NonTg mice (**upper panels**) at 1, 12, and 19 months of age. Lateral ventricles are outlined in red. (**B**) Quantification of lateral ventricular volumes is shown in the graphs. MRI analyses were performed in 7–9 NonTg and 5–9 Sph-1 KO mice. Data are presented as mean ± SEM. Statistical analysis was performed using unpaired two-tailed Student’s *t*-test; *** *p* < 0.0004 (1 and 12 months) and **** *p* < 0.0001 (19 months). (**C**) The extent of ventricle enlargement in Sph-1 KO mice relative to NonTg controls does not change significantly across ages. (**D**) Coronal brain sections from postnatal day 18 (p18) mice were stained with cresyl violet acetate (Nissl) at +0.26 mm from Bregma. Representative images from four mice demonstrate enlarged ventricles in Sph-1 KO mice (**right**) compared with NonTg controls (**left**). Scale bar, 1000 µm. Graph shows the lateral ventricle area in Sph-1 KO mice (*n* = 4) relative to NonTg controls (*n* = 4). Data are presented as mean ± SEM. Statistical analyses were performed using Student’s unpaired two-tailed *t*-test (* *p* ≤ 0.05).

Next, we analyzed the brains of the mice using magnetic resonance imaging (MRI) and found that all Sph-1 KO mice exhibited hydrocephalus at every age examined (1, 12, and 19 months) (Figure 6A,B). To determine whether hydrocephalus progresses with age, we quantified the percentage change in ventricular enlargement from 1 to 19 months. We observed an approximately two-fold increase in lateral ventricle volume across this age range (Figure 6C). In addition, Nissl staining of brains from 18-day-old pups revealed that Sph-1 KO mice already displayed clear enlargement of the lateral ventricles (Figure 6D), indicating that hydrocephalus arises early in the absence of synphilin-1.

**Figure 7:**
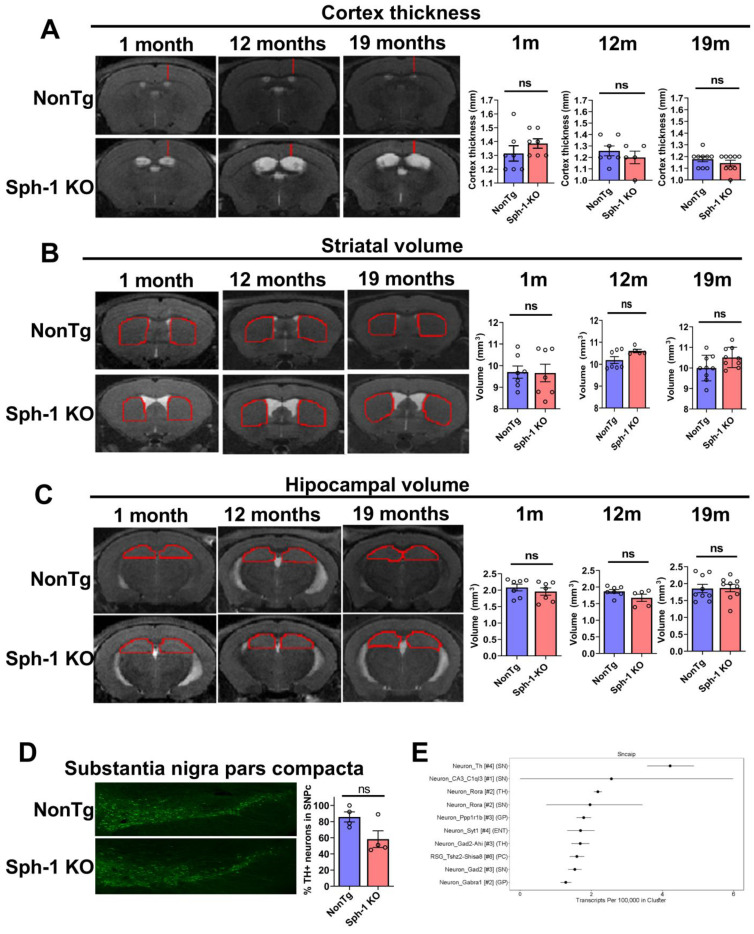
**Synphilin-1 knockout does not alter brain morphometry or cause nigral dopaminergic degeneration in adult mice.** As shown in Figure 6, a schematic timeline depicts the MRI scanning time points. (**A**–**C**) MRI-based quantification of cortical thickness (**A**), striatal volume (**B**), and hippocampal volume (**C**) in Sph-1 KO and NonTg mice across age (1–19 months). Representative images show the measured region in red; striatum and hippocampus were quantified from three matched slices per animal at the same bregma level. MRI analyses were performed in 7–9 NonTg and 5–9 Sph-1 KO mice. Data are mean ± SEM; unpaired two-tailed Student’s *t*-test; ns, *p* > 0.05. (**D**) Tyrosine hydroxylase (TH) immunohistochemistry-based counts of dopaminergic neurons in the substantia nigra at 16 months show no significant loss in Sph-1 KO mice (mean ± SEM; unpaired two-tailed Student’s *t*-test; ns, *p* > 0.05). Graph shows the % TH neurons in substantia nigra pars compacta in Sph-1 KO mice (*n* = 4) relative to NonTg controls (*n* = 4). (**E**) Dropviz RNA-seq (SNCAIP) cell-type clustering indicates enriched synphilin-1 expression in TH-positive substantia nigra neurons.

Hydrocephalus has been linked to neurodegenerative diseases such as AD and Dementia with Lewy bodies [29]. In addition, idiopathic normal pressure hydrocephalus (iNPH) can lead to motor dysfunction and cognitive impairment [30]. Therefore, we examined whether the hydrocephalus observed in Sph-1 KO mice could be associated with some degree of neurodegeneration. To address this, we performed MRI scans to assess the volume of different brain regions of Sph-1 KO. We measured cortical thickness as well as striatal and hippocampal volumes across ages 1 to 19 months and found no significant differences between Sph-1 KO and NonTg mice (Figure 7A–C).

Most importantly, we investigate whether synphilin-1 knockout leads to neurodegeneration in the substantia nigra pars compacta (SNpc). Because the SNpc cannot be reliably isolated by MRI, we performed immunohistochemistry on brain sections using anti-tyrosine hydroxylase (TH) antibody. In 16-month-old mice, Sph-1 KO mice showed a 32% reduction in TH-positive neurons in SNpc compared with NonTg controls; however, this decrease did not reach statistical significance (Figure 7D). This modest reduction is consistent with the absence of a motor phenotype (Figure 5), as motor impairment typically requires a loss of at least ~60% of TH neurons [31]. Notably, the observed trend toward reduced TH-positive neuron survival is consistent with the relatively high expression of synphilin-1 in SNpc dopaminergic neurons (Figure 7E) [32].

### 2.5. Synphilin-1 Is Essential for Maintaining Microtubule Homeostasis in Ependymal Cells

**Figure 8:**
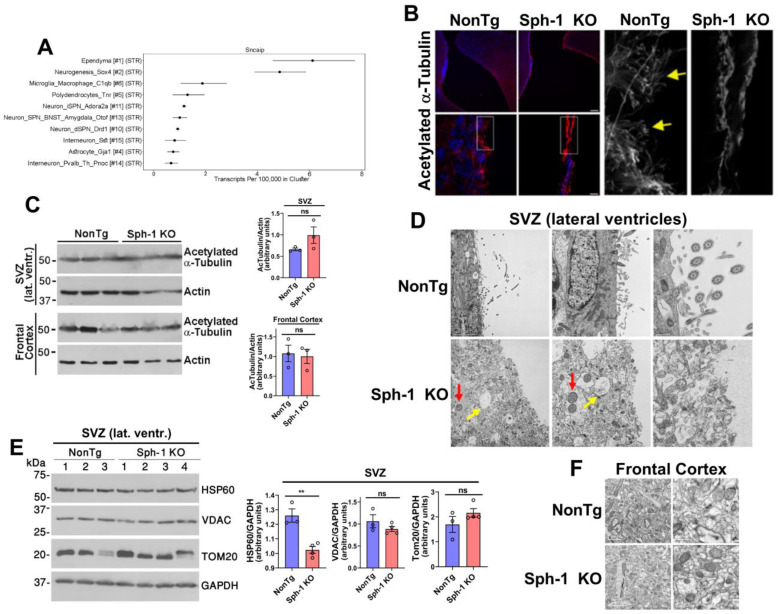
**Synphilin-1 knockout causes ependymal cilia loss.** (**A**) Dropviz RNA-seq cell-type clustering of SNCAIP (synphilin-1) shows enriched expression in ependymal cells. (**B**) Immunohistochemistry of 15-month brain sections stained for acetylated α-tubulin (cilia; red) and Hoechst (nuclei; blue) shows reduced ependymal cilia in Sph-1 KO compared with NonTg controls. Insets (boxed regions) highlight cilia morphology; yellow arrows mark intact cilia in NonTg. Scale bars: 100 µm (**top**) and 10 µm (**bottom**). (**C**) Acetylated α-tubulin levels in SVZ and frontal cortex from 20-month mice assessed by immunoblot. The image is representative of three independent Western blot analyses for both SVZ and frontal cortex. Graphs to the right depict the relative levels of acetylated α-tubulin in Sph-1 KO mice compared with NonTg controls. For each group, 3 animals were analyzed. Data are presented as mean ± SEM; unpaired two-tailed Student’s *t*-test; ns, *p* > 0.05. (**D**) TEM of SVZ/lateral ventricle at 12 months shows organized, ciliated ependyma with normal ciliary ultrastructure and mitochondria in NonTg, versus disorganized ependyma with cilia loss, cellular edema (red arrows), and rounded mitochondria (yellow arrows) in Sph-1 KO. Scale bars: 5, 2, and 0.5 µm. (**E**) Levels of indicated mitochondrial protein in SVZ from 20-month mice assessed by immunoblot. The image is representative of three independent Western blot analyses. Graphs to the right depict the relative levels of mitochondrial proteins in Sph-1 KO mice compared with NonTg controls. For each group, 3–4 animals were analyzed. Data are presented as mean ± SEM; unpaired two-tailed Student’s *t*-test; ** *p* < 0.01 for HSP60 and ns, *p* > 0.05 for VDAC and Tom20. (**F**) TEM of frontal cortex at 12 months shows no detectable mitochondrial differences between genotypes. Scale bars: 1 µm (**left**) and 200 nm (**right**). Panels (**B**,**D**,**F**) show representative images from three Sph-1 KO and three NonTg mice.

We next sought to investigate the mechanisms underlying the development of hydrocephalus in Sph-1 KO mice. Because ciliated ependymal cells play a critical role in cerebrospinal fluid (CSF) homeostasis [33], and because the synphilin-1 gene SNCAIP is enriched in these cells (Figure 8A) [32], we next examined their morphology and organization in Sph-1 KO brains. Immunohistochemical analysis was performed using acetylated α-tubulin as a marker of microtubule-rich cilia [34]. At low magnification, acetylated α-tubulin labeled appeared more intense in the ependymal layer of Sph-1 KO mice (Figure 8B, upper panels). However, higher magnification revealed a marked loss of cilia in Sph-1 KO ependymal cells, accompanied by an abnormal accumulation of acetylated α-tubulin in the cytoplasm, in contrast to the well-organized cilia observed in NonTg controls (Figure 8B, lower panels and insets). Moreover, Western blot analysis showed that total levels of acetylated α-tubulin were unchanged in the subventricular zone of Sph-1 KO mice compared with NonTg controls, and were similarly unchanged in the frontal cortex (Figure 8C). These findings suggest that synphilin-1 is not required for global tubulin acetylation but may instead be necessary for the proper stability and organization of microtubules in ependymal cells.

Based on these observations, we performed transmission electron microscopy and found that ependymal cells lining the lateral ventricles of 12-month-old Sph-1 KO mice were disorganized and lacked cilia compared with NonTg controls (Figure 8D). In addition, the subventricular zone of Sph-1 KO mice showed fluid accumulation and disrupted subcellular architecture (Figure 8D). Mitochondria within this region were also abnormally enlarged and rounded in Sph-1 KO mice (Figure 8D). Interestingly, consistent with the frontal cortex (Figure 2B), the mitochondrial matrix chaperone HSP60 was also reduced in the subventricular zone of Sph-1 KO mice (Figure 8E).

In contrast, no structural abnormalities were detected in the frontal cortex of Sph-1 KO mice, and cortical mitochondria structure appeared unaffected by synphilin-1 loss (Figure 8F). Together, these findings indicate that synphilin-1 is essential for maintaining ependymal cell integrity, likely by supporting microtubule stability, and further suggest that synphilin-1 regulates a key protein involved in mitochondrial proteostasis across the brain.

## 3. Discussion

Synphilin-1 was first identified as an α-synuclein-interacting protein [5]. Subsequent work in cell culture and in vivo models showed that synphilin-1 promotes α-synuclein aggregation [5,10,11]. Synphilin-1 was also detected as a component of Lewy bodies [8]. Beyond these pathological links, synphilin-1 has also been proposed to exert protective effects [10,14,19,20], yet its physiological function remains unresolved. To define the in vivo role of synphilin-1, we generated a synphilin-1 knockout (Sph-1 KO) mouse line (Figure 1). Synphilin-1 deletion did not affect body weight or survival (Figure 3), nor did it produce overt deficits in motor performance (Figure 4 and Figure 5), learning, or anxiety-related behaviors (Figure 5). However, older Sph-1 KO mice showed a mild trend toward dopaminergic degeneration (Figure 7), consistent with the relatively high synphilin-1 expression in the substantia nigra. Western blot analysis revealed no changes in the abundance of α-synuclein, synaptic proteins, or outer membrane mitochondrial proteins following synphilin-1 loss (Figure 2). In contrast, levels of the matrix chaperone HSP60 were reduced throughout the brain (Figure 2 and Figure 8), suggesting that synphilin-1 contributes to mitochondrial proteostasis. Structurally, synphilin-1 knockout did not alter cortical, striatal, or hippocampal volumes (Figure 7). Strikingly, Sph-1 KO mice developed early-onset hydrocephalus (Figure 6), accompanied by pronounced collapse and disorganization of ciliary microtubules and abnormal mitochondrial morphology in ependymal cells lining the ventricles (Figure 8), which also express high levels of synphilin-1. Collectively, these findings indicate that synphilin-1 is required to preserve ependymal cytoskeletal integrity and maintain mitochondrial proteostasis across the brain, with broader implications for CSF homeostasis, neuronal function, and our understanding of synphilin-1’s role in PD.

Synphilin-1 has been repeatedly proposed to be cytoprotective across diverse experimental contexts [10,14,19,20], yet the mechanistic basis for this effect has remained unclear. Our observation that synphilin-1 deletion reduces the levels of the mitochondrial matrix chaperone HSP60 (Figure 2 and Figure 8) provides a potential link between these prior reports and mitochondrial homeostasis. HSP60 is a central component of mitochondrial protein quality control, supporting the folding and maintenance of matrix proteins and thereby limiting the accumulation of misfolded or damaged proteins that can trigger mitochondrial dysfunction and cellular stress [23,35]. A reduction in HSP60 following synphilin-1 loss therefore suggests that synphilin-1 may help sustain mitochondrial proteostasis, preserving mitochondrial integrity under baseline conditions and potentially buffering cells against proteotoxic and oxidative challenges. In this framework, the previously described that the cytoprotective actions of synphilin-1 could reflect, at least in part, its capacity to maintain mitochondrial protein quality control, thereby reducing vulnerability to stressors that are particularly relevant to neurodegeneration.

We previously demonstrated that synphilin-1, together with PINK1, supports basal mitophagy in cellular models [15]. We therefore examined whether synphilin-1 deletion in vivo leads to mitochondrial accumulation due to impaired turnover. Although we did not detect an overall increase in mitochondrial protein levels in synphilin-1 knockout mice (Figure 2 and Figure 8), we observed prominent swollen mitochondria in ependymal cells of Sph-1 KO mice (Figure 8), suggesting that mitophagy may be selectively compromised in this specific brain region where synphilin-1 is highly expressed. Further work will be required to clarify the contribution of synphilin-1 to mitophagy in vivo, including more detailed assessments of mitochondrial turnover in constitutive knockout mice and analysis of mitochondrial content in conditional synphilin-1 knockout models.

The involvement of synphilin-1 in hydrocephalus has not, to our knowledge, been reported previously. Here, we show that synphilin-1 deletion leads to marked hydrocephalus (Figure 6). Hydrocephalus can result from increased CSF production or from impaired ciliary motility, which reduces CSF flow and promotes ventricular CSF accumulation. Given the pronounced collapse of ventricular cilia in synphilin-1-deficient mice (Figure 8), impaired ciliary function is the most plausible driver of the hydrocephalus observed. Because hydrocephalus appears early in development, consistent with constitutive loss of synphilin-1 from embryonic stages, it remains uncertain whether synphilin-1 deficiency primarily interferes with ciliogenesis or instead destabilizes cilia shortly after they form. Both mechanisms are supported by examples in the literature. For example, deletion of the endosomal trafficking protein SNX27 or the RNA-binding protein HuR impairs ependymal cell differentiation and leads to hydrocephalus [36,37]. Conversely, Kif6 knockout results in progressive degeneration of ependymal cilia and reduced ciliary motility over time [38]. Future studies using conditional synphilin-1 knockout models will be essential to determine whether synphilin-1 acts predominantly in the formation or maintenance of ependymal cilia. Regardless of the precise mechanism, these findings identify synphilin-1 as an important regulator of ependymal cilia integrity and CSF homeostasis.

Deletion of several proteins essential for primary cilia homeostasis has also been associated with hydrocephalus in mice, such as IFT88 and KIF3A/KIF3B [39]. However, because primary cilia also participate in different signaling pathways, mouse models with impaired primary ciliary function often exhibit additional embryonic abnormalities, such as neural tube defects, altered brain patterning, polycystic kidney disease, and skeletal malformation [40,41,42]. Although the synphilin-1 knockout mice did not show any overt abnormalities beyond hydrocephalus (Figure 6), further studies will be necessary to determine whether synphilin-1 contributes to primary cilia formation and function.

In addition to the collapse of cilia in ventricular ependymal cells, we also observed profound disruption of their intracellular architecture (Figure 8), further underscoring the essential role of synphilin-1 in ciliary brain cells. Notably, although cytoskeletal protein expression remained unchanged, electron microscopy revealed a loss of recognizable organelles and markedly enlarged, rounded mitochondria in the absence of synphilin-1 (Figure 8). These abnormalities are consistent with the proposed cytoskeletal functions of synphilin-1, inferred from its multidomain structure and its enriched expression in progenitor populations [5,32].

Ependymal cells contribute to the formation and maintenance of the blood–brain barrier, helping to restrict the unregulated entry of solutes into the brain while also supporting the clearance of metabolic waste [43]. More specifically, studies show dysfunction of the blood–brain barrier in PD [44,45]. Therefore, our findings that synphilin-1 is required to preserve ependymal cell integrity, including normal cilia architecture (Figure 8), and that its loss leads to CSF accumulation (Figure 6) are consistent with the possibility that synphilin-1 contributes to the maintenance of barrier functions relevant to PD, including the blood–brain barrier.

Our findings uncover an unexpected requirement for synphilin-1 in maintaining cytoskeletal and organelle homeostasis in specialized ciliated brain epithelia and suggest broader effects on mitochondrial quality control. These results broaden our understanding of synphilin-1 biology in the nervous system and provide a new context for its potential relevance to PD.

## 4. Materials and Methods

### 4.1. Animals

All animal experiments and procedures were conducted in accordance with the guidelines of the Committee for the Supervision of Animal Experiments (Technion-Israel Institute of Technology). All efforts were made to minimize animal suffering and to reduce the number of animals used. Any animal exhibiting signs of distress or losing more than 10% of its body weight was promptly evaluated, including assessment for pain, to identify the cause and minimize stress. Any mice assessed as unwell were excluded from all analyses. All experimental procedures were approved by the Institutional Committee for Animal Care and Use at the Technion-Israel Institute of Technology, Faculty of Medicine, Haifa, Israel (approvals no. IL164112017 and IL0150124). C57BL/6N-Sncaip^tm1b(EUCOMM)Wtsi^ (EM:08403) synphilin-1 knockout mice were obtained from the European Mouse Mutant Cell Repository (EUCOMM). This line was generated from the Sncaip^tm1a^ allele, in which the critical exon (exon 4) was flanked by loxP sites and a LacZ reporter cassette inserted at the targeted locus. Cre-mediated recombination excised exon 4 together with the floxed cassette, producing the Sncaip^tm1b(EUCOMM)Wtsi^ allele in which exon 4 was deleted, and LacZ remained as a reporter of endogenous SNCAIP expression (Figure 1A). Heterozygous Sncaip^tm1b(EUCOMM)Wtsi^ mice obtained from EUCOMM were intercrossed to generate homozygous Sncaip ^tm1b(EUCOMM)Wtsi^ mice, referred to here as Sph-1 KO mice. Control groups consisted of non-transgenic littermates, i.e., siblings from the Sncaip^tm1b(EUCOMM)Wtsi^ breeding colony that do not carry the Sncaip^tm1b(EUCOMM)Wtsi^ allele. When needed, wild-type mice were purchased from Harlan Laboratories Israel Ltd. Jerusalem, Israel). A total of 151 Sph-1 KO and NonTg mice were used in this study. Mice were housed (2–5 mice per cage) under pathogen-free, controlled environmental conditions (temperature 21 ± 2 °C; relative humidity 55 ± 5%) with a 12 h light/dark cycle (lights on at 7:00 AM). Mice had ad libitum access to food and water.

Genotyping was performed using polymerase chain reaction (PCR). Genomic DNA was extracted from tail biopsies as described [46] and used for PCR amplification with the following primers: 5′-CACACCTCCCCCTGAACCTGAAAC-3′ and 5′-ATCTCAATCCAACTGGCGCACACT 3′ (322 bp relative to the knockout allele) plus 5′-CGCCCAGTGAAGCGAGTTTCTCCATG-3′ and 5′-GCCACATTTACCCAGAGCTGGCGAGCTCTC-3′ (513 bp relative to exon 4 of wild-type synphilin-1).

### 4.2. Western Blot Analysis

Brain tissues from 20-month-old synphilin-1 knockout mice and age-matched NonTg littermates were dissected and homogenized in buffer containing 50 mM of Tris-HCl (pH 7.4), 140 mM of NaCl, 30 mM of MG132, 2 mM of Na_3_VO_4_, 200 mM of NaF, 10 mM of PPi, 20 mM of β-Glycerol phosphate, and a cocktail of protease inhibitors (Mini-complete, Roche, Penzberg, Germany). After sonication for 30 s, 1% triton X-100 and 0.1% sodium dodecyl sulfate (SDS) were added to the samples. The samples were incubated for 15 min at 4 °C and then cleared by centrifugation at 13,000× *g* for 5 min at 4 °C. After the addition of loading buffer, lysates were denatured at 100 °C for 5 min, separated on 11% SDS-polyacrylamide gels, and transferred to 0.20 µm cellulose nitrocellulose membranes [13].

Membranes were probed with the following primary antibodies: mouse anti-actin (691001, MP Biomedical, Irvine, CA, USA), rabbit anti-GAPDH (G9545, Sigma, St. Louis, MO, USA), mouse anti-α-synuclein (610787, BD Biosciences, San Jose, CA, USA), mouse anti-PSD95 (SMS-122, StressMarq, Victoria, BC, Canada), mouse anti-synaptophysin (sc-17750, Santa Cruz Biotechnologies, Dallas, TX, USA), mouse anti-HSP60 (sc-376261, Santa Cruz Biotechnologies, Dallas, TX, USA), rabbit anti-TOM20 (sc-11415, Santa Cruz Biotechnologies, Dallas, TX, USA), and rabbit anti-VDAC (4662s, Cell Signaling, Danvers, MA, USA). Rabbit polyclonal anti-synphilin-1 was generated and purified as previously described [5]. Blots were developed with secondary anti-rabbit or anti-mouse antibodies conjugated to peroxidase (Jackson Laboratory) and reacted with ECL Dura (Thermo Scientific, Waltham, MA, USA). The numbers shown in the Western blot figures indicate the number of animals (biological replicates) analyzed. Western blot analyses were performed using samples from twelve mice.

### 4.3. Behavioral Assessment

Behavioral tests were performed on both male and female neonatal and adult mice. Neonatal assessments included ambulation, surface righting, negative geotaxis, front-limb suspension, hindlimb suspension, and grip strength, conducted on postnatal day 10 (P10) as previously described [26,47]. Behavioral testing of pups was performed on fifteen mice. Adult behavioral testing included the pole test, cylinder test, rotarod, open field, Morris water maze (MWM), and the startle and auditory sustained attention test (ASAT). Behavioral testing of mice older than one month was performed on forty-nine mice.

### 4.4. Ambulation Test

Ambulation in neonatal mice was assessed by observing spontaneous walking and crawling behavior. Pups were placed individually on a regular padded bench surface and monitored simultaneously from both top and side views. Each pup was allowed to move freely for a period of 3 min. Ambulation was scored using the following scale: 0 = no movement, 1 = crawling with asymmetric limb movement, 2 = slow crawling with symmetric limb movement, and 3 = fast crawling or walking [47].

### 4.5. Surface Righting Test

The surface righting test was used to assess the righting reflex; a motor reflex was defined as the ability of a mouse pup to reorient itself from a supine to a prone position. Pups were placed on their backs on a regular padded bench surface and gently held in position for 5 s. Upon release, the latency to return to the prone position was recorded in seconds [47]. Each trial was terminated after a maximum duration of 60 s if the pup failed to right itself.

### 4.6. Negative Geotaxis Test

The negative geotaxis test was used to assess motor coordination and vestibular function in mouse pups. When placed facing downward on an inclined surface, pups are expected to reorient themselves upward in response to gravitational cues. Pups were positioned head-down on a 45° incline and gently held in place for 5 s. Upon release, the latency to turn and orient upward was recorded in seconds [47]. Each trial was terminated after a maximum duration of 120 s if the pup failed to reorient.

### 4.7. Front-Limb Suspension Test

Front-limb strength in mouse pups was assessed using the front-limb suspension test. Pups were gently supported by the body and allowed to grasp a homemade metal wire stretched across the opening of a transparent glass beaker (14 cm in height, 10 cm in diameter) [47]. The bottom of the beaker was padded to minimize injury in the event of a fall. Upon release, the latency to fall was recorded in seconds using a timer.

### 4.8. Hindlimb Suspension Test

Hindlimb strength was assessed using the hindlimb suspension test. A standard 50 mL conical tube with a padded bottom was used. Pups were gently held and positioned face down in the tube, with the hindlimbs hanging freely over the rim. Upon release, the latency to fall was recorded in seconds using a timer [47].

### 4.9. Grip Strength Test

This test was used to assess the combined strength of all four paws in mouse pups. Pups were placed on a regular wire mesh in a horizontal position and allowed to acclimate for 5 s. The mesh was then slowly rotated from a horizontal to a vertical orientation. The angle of the mesh at which the pup released its grip and fell was recorded. If a pup maintained its grip until the mesh reached a fully inverted position (180°), the latency to fall was recorded in seconds using a timer [47].

### 4.10. Pole Test

The pole test was used to assess motor function, as previously described [27]. The homemade apparatus consisted of a wooden base (25 cm × 25 cm × 4 cm), a lightly perforated wooden pole (50 cm high, 1 cm in diameter) to prevent slipping, and a plastic ball (2 cm in diameter) mounted at the top of the pole. The apparatus was placed inside the animals’ home cage, with the base covered in bedding to minimize potential injury from falls. Mice were trained for two consecutive days (3 sessions per day) to descend the pole after being placed on its top. On the third day, testing was performed with three trials per mouse, separated by 3 min intervals. The time required for each mouse to descend the pole and return to the home cage was recorded.

### 4.11. Cylinder Test

The cylinder test was used to assess motor asymmetry and forelimb and hindlimb motility, as previously described [48]. Animals were habituated to the testing area for at least 30 min prior to testing. The testing area and equipment were cleaned with 70% ethanol and allowed to dry between animals. Mice were placed individually in a regular transparent glass cylinder (14.5 cm high, 9.5 cm in diameter). Over a 2 min observation period, the total number of rearing events involving forelimb contact with the cylinder wall was recorded, regardless of left or right paw use. A rear was defined as the mouse lifting its forelimbs and making contact with the cylinder wall using one or both forelimbs. A new rear was scored only after the mouse withdrew from the wall and returned all limbs to the bottom of the cylinder. To prevent habituation, each mouse was tested only once.

### 4.12. Rotarod

To assess motor function, coordination, and motor learning, the rotarod test was performed using a rotarod apparatus (San Diego Instruments, San Diego, CA, USA) as previously described [28]. The apparatus consists of a four-lane rotating rod made of non-reflective black Persplex, separated by opaque black dividers. For animal safety, the apparatus is enclosed by a sawdust-filled landing chamber (45.7 cm high) to cushion falls. A red infrared beam system was used for the automatic recording of latency to fall (in seconds). Mice were acclimated to the apparatus for 60 s prior to testing. Each session consisted of four consecutive trials, with a 2 min rest period between trials. For each trial, the rotarod was set to an initial speed of 5 revolutions per minute (rpm) for 15 s, followed by constant acceleration at a rate of 0.1 rpm per second (maximum speed 50 rpm after 465 s), and the fall latency for each mouse was measured. The average fall latency for both groups was shown on the first and last day of the trials.

### 4.13. Open Field Test

Locomotor activity and exploratory behavior were assessed using the open field test. Mice were placed in a black, non-reflective Perspex arena (50 × 50 × 40 cm) with a white, non-reflective Perspex floor. The arena was located in a dimly lit room. Each mouse was placed in a corner of the arena facing the wall and allowed to freely explore the arena for 5 min. Animal behavior was recorded using a uEye UI-308x-CP-C camera (IDS Imaging Development Systems GmbH, Obersulm, Germany) and analyzed with EthoVision XT 11.5 software (Noldus, Wageningen, The Netherlands), as previously described [28,48].

### 4.14. Morris Water Maze

Spatial learning and memory were assessed using Morris water maze, as previously described [28]. The apparatus consisted of a circular white plastic tank (1.4 m in diameter, 0.6 m high) filled with water to a depth of 45 cm and maintained at 23 ± 1 °C. A circular escape platform (10 cm in diameter) was submerged 1 cm below the water surface and was not visible to the animals. Visual cues were provided only by external landmarks positioned on the surrounding walls. Training was conducted over six consecutive days, with four trials per day and a 3 min inter-trial interval. At the start of each trial, mice were randomly placed in the maze facing the wall at one of four starting positions (north, south, east, or west) and allowed to swim freely until locating the hidden platform or until a maximum of 90 s had elapsed. At the end of each trial, mice were either guided to or allowed to remain on the platform for 15 s. On day six, the hidden platform was removed, and a probe trial was conducted. Mice were allowed to swim freely for 60 s, and spatial memory was assessed by measuring time spent in each of the four virtual quadrants, with particular emphasis on the target quadrant. The swimming and behavior of the mice were recorded using a uEye UI-308x-CP-C camera (IDS Imaging Development Systems GmbH, Obersulm, Germany) and analyzed with EthoVision XT 11.5 software (Noldus, The Netherlands), as previously described [28].

### 4.15. Startle Response and Auditory Sustained Attention Test (ASAT)

Acoustic startle response and auditory sustained attention were assessed using a sound-attenuated and ventilated chamber (Kinder Scientific, Poway, CA, USA). Following a 3 min acclimatization period, a continuous background mouse of 57 dB was presented throughout the session. The test session consisted of 144 trials delivered in a pseudorandom order, with a mean inter-trial interval of 8.4 s (range: 6–12 s). Startle responses were elicited by 30 ms acoustic pulse-alone stimuli presented at ten intensities in 6 dB increments (60, 66, 72, 78, 84, 90, 96, 102, 108, 114, 120 dB), with eight trials per intensity. Pre-pulse inhibition (PPI) trials consisted of a 120 db startle pulse preceded by an 80 ms interval by a 20 ms pre-pulse at intensities of 2, 4, 8, 12, 16, or 21 dB above the background noise (i.e., 59, 61, 65, 69, 73, or 78 dB), with eight trials per pre-pulse intensity. In addition, eight no-stimulus trials were included, during which no acoustic stimulus was presented. ASAT performance was calculated based on pre-pulse inhibition using the following formula: ASAT (%) = 100 − [(maximum response during pre-pulse trials/maximum response during pulse-alone trials) × 100], representing the percentage of response inhibition [49]. The probability of a startle response was determined as previously described [28,50]. Briefly, the maximal response during no-stimulus trials was recorded, and the mean (μ) and standard deviation (σ) were calculated. Baseline noise was estimated using the coefficient of variation (CV = σμ). A valid startle response was defined as a response amplitude exceeding three times the noise level (3 × CV).

### 4.16. MRI and Image Analysis

MRI scans were performed on eighteen mice, as previously described [51]. Briefly, scans were done using a 9.4T horizontal MRI system (Bruker Biospec, Ettlingen, Germany) equipped with a cylindrical volume coil (86 mm inner diameter) for RF transmission and a 20 mm surface coil for signal reception. During imaging, animals were anesthetized with 1–2% isoflurane in oxygen (0.7 L/min). Respiration was continuously monitored (Small Animal Instruments, Stony Brook, NY, USA), and body temperature was maintained using circulating warm water.

Anatomical T2-weighted brain images were acquired using a Rapid Acquisition with Relaxation Enhancement (RARE) sequence with the following parameters: slice thickness 0.4 mm, 31 slices, in-plane resolution 100 µm; TR = 4000 ms, TE = 36 ms, RARE factor = 12, field of view (FOV) = 1.6 × 1.6 mm^2^, matrix size = 160 × 160, and 6 averages. The total scan time was 5.5 min.

Image processing and volumetric analyses were performed using the Medical Image Processing, Analysis, and Visualization (MIPAV) software package (NIH; http://mipav.cit.nih.gov). Measurements for calculating specific brain volumes were done by segmenting the lateral ventricles at −0.30, −0.70, and −1.1 mm from Bregma and the hippocampus at −2.06 mm to −1.58 mm from Bregma (according to Paxinos and Franklin *The Mouse Brain in Stereotaxic Coordinates* [52]). Cortical thickness (at −0.70 mm from Bregma) and striatum volume (at 0.14 mm to 0.94 mm from Bregma) were also quantified using MIPAV software (Version 11.3.3). Average regional volumes were calculated for each experimental group.

### 4.17. Brain Perfusion and Cryosection

Following completion of behavioral testing and MRI scanning, fourteen mice were perfused and cryosectioned as previously described [27]. Briefly, mice were deeply anesthetized with isoflurane and then received an intraperitoneal injection of ketamine/xylazine. Once a surgical plane of anesthesia was confirmed (absence of pedal withdrawal reflex), mice were transcardially perfused via the left ventricle with ice-cold phosphate-buffered saline (PBS) to clear the blood, followed immediately by 4% paraformaldehyde (PFA; pH 7.4) for tissue fixation. Brains were carefully dissected and post-fixed in 4% PFA, then cryoprotected by immersion in increasing concentrations of sucrose in PBS until the tissue sank. For cryosectioning, brains were embedded in optimal cutting temperature (OCT) compound, rapidly frozen, and stored at −80 °C until sectioning. Coronal sections (25 µm) were cut on a Leica CM3050S cryostat (Nußloch, Germany), and striatal and nigral sections were collected as free-floating sections and stored in PBS at 4 °C until subsequent immunohistochemical analysis.

### 4.18. Immunohistochemical Analysis

Brain sections were processed for free-floating immunohistochemistry. Briefly, sections were blocked in 5% normal goat serum (Jackson’s Laboratory, Farmington, CT, USA) in PBS containing 0.1% Triton X-100 for 1 h at room temperature. Rabbit anti-tyrosine hydroxylase 1:500 (AB152, Millipore, Burlington, MA, USA) and mouse anti-acetylated α-tubulin 1:500 antibodies (sc-23950, Santa Cruz Biotechnologies, Dallas, TX, USA) were incubated overnight at 4 °C. After washing, sections were then incubated with Alexa Fluor 488- or Cy3™-conjugated secondary antibodies (Jackson’s Laboratory), followed by counterstaining with the nuclear marker Hoechst 33342 (Thermo Scientific). Stained sections were mounted onto SUPERFROST^®^ PLUS slides (Thermo Scientific) using SHANDON Immu-Mount (Thermo Scientific) and stored at 4 °C in a light-protected box until analysis [51].

For visualization of ependymal cells and cilia, sections from six mice were imaged using AiryScan super-resolution on a Zeiss LSM 880 confocal microscope (Jena, Germany). Optical sections were acquired with 10× and 63× immersion objectives at a resolution of 1024 × 1024 pixels. All pictures were captured using identical laser settings and magnification parameters [15].

For nigral dopaminergic analysis, TH-stained sections from eight mice were scanned using an Olympus SLIDEVIEW VS200 microscope (Evident Scientific, Waltham, MA, USA). TH-positive neurons in the substantia nigra pars compacta (SNpc) were quantified in three adjacent sections per mouse using ImageJ. Counts were averaged across sections for each mouse and expressed as the percentage of TH-positive neurons relative to the NonTg control group [27].

### 4.19. Nissl Staining

Striatal 25 µm thick sections from eight mice were washed with PBS, mounted onto SUPERFROST^®^ PLUS slides, and stained with cresyl violet acetate (Thermo Scientific) [53]. Slides were dehydrated through graded ethanol solutions, cleared in xylene, and coverslipped using DPX mounting medium (Sigma-Aldrich, St. Louis, MO, USA). Images of Nissl-stained sections were acquired using a widefield Zeiss inverted microscope (Jena, Germany).

### 4.20. Slide Scanning

Images of tyrosine hydroxylase-stained substantia nigra sections from eight mice were acquired using a Pannoramic 250 Flash III automated digital slide scanner (3D Histech Ltd., Budapest, Hungary). Fluorescence imaging was performed with a 20×/0.8 Plan Apochromat objective using LED 475/28 illumination and a Cy2 filter set (excitation 485/26 nm, emission 521/27 nm) as well as LED 386/23 illumination with a DAPI filter set (excitation 387/18 nm, emission 440/44 nm). Cells were counted using ImageJ version 1.53C [27].

### 4.21. Electron Microscopy

Brain slices from six mice were fixed in a solution containing 2% glutaraldehyde and 2% paraformaldehyde in 0.1 M sodium cacodylate buffer (Electron Micrsocopy Sciences, Hatfield, PA, USA). Following post-fixation with 1% osmium tetraoxide, samples were stained with 1% uranyl acetate (Electron Microscopy Sciences). Tissues were then dehydrated through a graded ethanol series and embedded in Epon resin. Ultrathin sections (75 nm) were cut using an ultramicrotome (UC7 EM), mounted on copper grids, and examined with a Talos L120C transmission electron microscope operated at 120 kV (Thermo Scientific) [54].

### 4.22. Statistical Analysis

All statistical analyses were performed using GraphPad Prism version 8.0.8 (GraphPad Software, Inc., San Diego, CA, USA). Data are presented as mean ± standard error of the mean (SEM). The sample size (*n*) and the definition of *n* (e.g., biological replicates) for each experiment are provided in the corresponding figure legends. For experiments involving two independent variables, statistical significance was evaluated using two-way analysis of variance (ANOVA), followed by Tukey’s multiple-comparisons post hoc test. For comparisons between two independent groups, Student’s unpaired two-tailed *t*-test was used. All tests were two-sided, and differences were considered statistically significant at *p* ≤ 0.05.

## 5. Conclusions and Future Perspectives

Collectively, our findings demonstrate that synphilin-1 is required to preserve ependymal cell integrity and organize motile cilia that drive cerebrospinal fluid flow. The early-onset hydrocephalus observed in Sph-1 knockout mice, together with disrupted microtubule architecture and altered mitochondrial morphology, supports a model in which synphilin-1 coordinates cytoskeletal organization and organelle homeostasis in ciliated ependymal cells. Notably, synphilin-1 loss also reduces HSP60 levels, implicating synphilin-1 in broader regulation of mitochondrial proteostasis throughout the brain. Future work will help to define the molecular mechanisms through which synphilin-1 stabilizes microtubules and maintains mitochondrial homeostasis and determine how these processes influence α-synuclein pathology, neuroinflammation, and dopaminergic neuron vulnerability. Finally, testing synphilin-1 function in PD-relevant mouse models will clarify the extent to which impaired ciliogenesis and mitochondrial proteostasis connect synphilin-1 biology to PD.

## Data Availability

The original contributions presented in this study are included in the article/Supplementary Material. Further inquiries can be directed to the corresponding authors.

## Supplementary Materials

The following supporting information can be downloaded at https://www.mdpi.com/article/10.3390/ijms27083499/s1.

## Abbreviations

PD, Parkinson’s disease; KO, knockout; Sph-1, synphilin-1; SVZ, subventricular zone; TEM, transmission electron microscopy.
