# Supplementary material for: Synphilin-1 Is Essential for Cytoskeletal Integrity of Brain Ventricular Cilia and Mitochondrial Proteostasis
Source: Int J Mol Sci. 2026 Apr 14;27(8):3499. doi: 10.3390/ijms27083499 (PMC13116186; doi:10.3390/ijms27083499)
Supplement: Supplementary file 1 [file ijms-27-03499-s001.zip › ijms-4103158-supplementary.pdf]

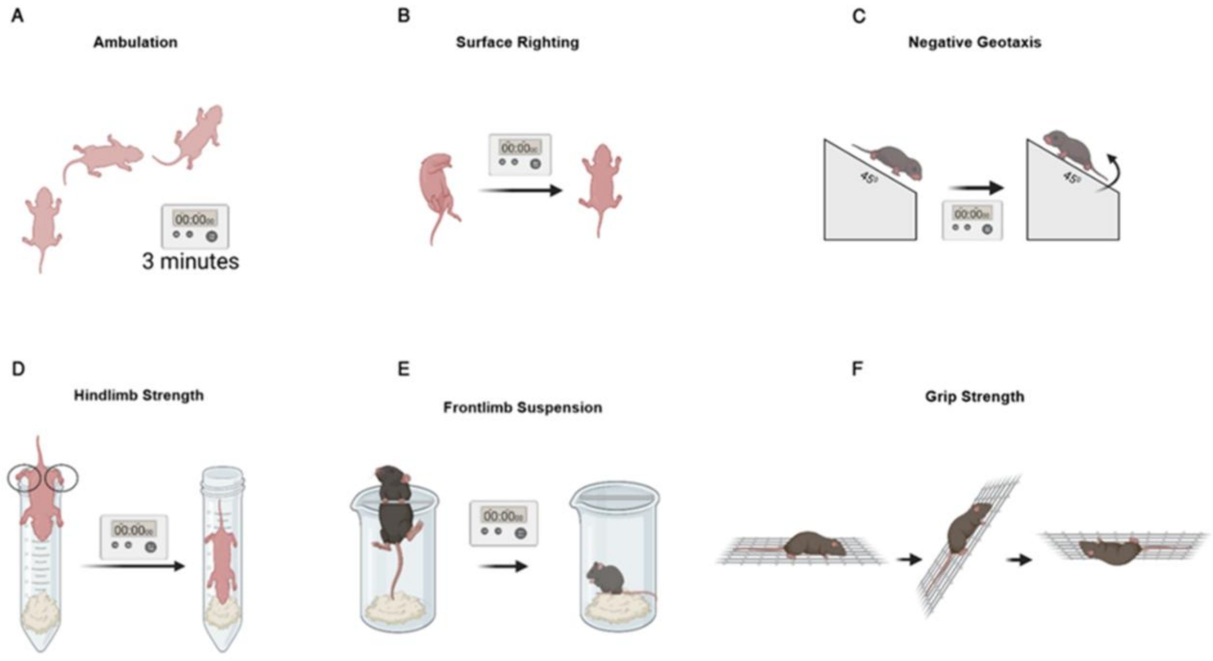


**Figure S1.** Schematic representations of neonatal behavioral tests. (**A**) The Ambulation test was conducted for 3 minutes to score pup crawling behavior according to a predetermined scale. (**B**) The Surface Righting test assesses the pup's ability to flip onto its feet from a supine position. (**C**) The Negative Geotaxis test evaluates motor coordination by measuring the pup's ability to orient and climb uphill on an inclined surface. (**D**) The Hindlimb Strength test measures hindlimb strength and neuromuscular function. (**E**) The Forelimb Suspension test evaluates forelimb strength based on the pup's ability to hang from a bar or ledge. (**F**) The Grip Strength test assesses the combined strength of all four limbs simultaneously.

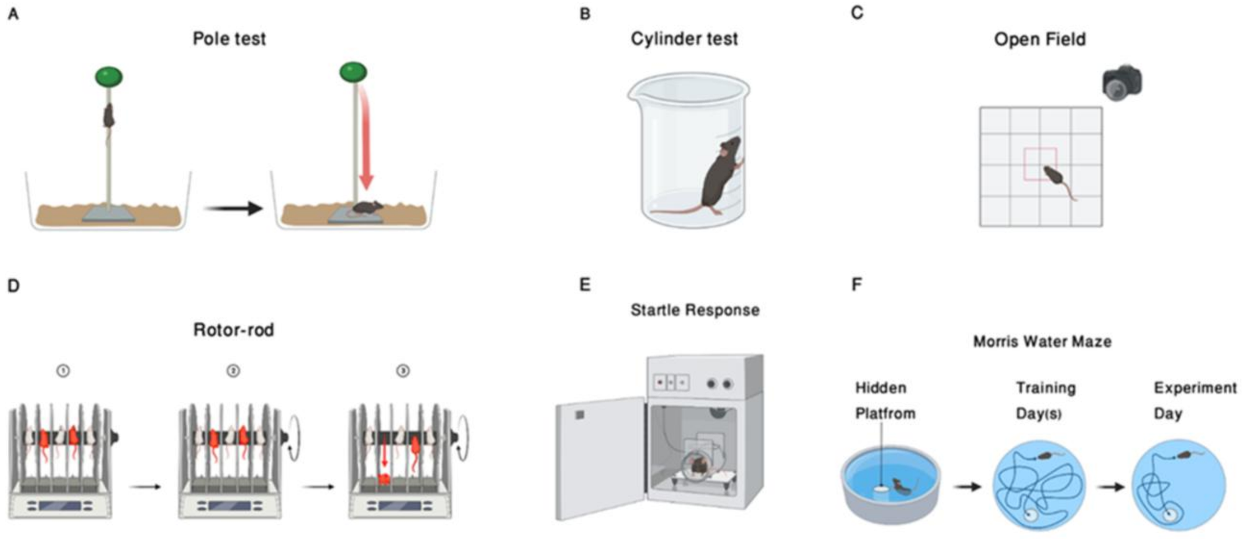


**Figure S2.** Schematic representations of adult behavioral tests. (**A**) The Pole test evaluates motor function and coordination. (**B**) The Cylinder test assesses motor asymmetry and the use of both forelimbs and hindlimbs. (**C**) The Open

Field test measures locomotor activity and anxiety-like behavior. (**D**) The Rotarod test evaluates motor coordination, balance, and motor learning. (**E**) The Startle Response test measures habituation and sensorimotor gating. (**F**) The Morris Water Maze (MWM) test assesses hippocampal-dependent spatial learning and memory.
